# Six-month efficacy and safety of oxymetazoline hydrochloride 0.1% in Japanese patients with acquired blepharoptosis: a phase 3 study

**DOI:** 10.1007/s10384-026-01340-5

**Published:** 2026-04-10

**Authors:** Hitoshi Ishikawa, Koji Oka, Hiroshi Inoue

**Affiliations:** 1 Orthoptics and Visual Sciences, Kitasato University School of Allied Health Sciences, Kanagawa, Japan; 2 Santen Pharmaceutical Co., Ltd, 4-20 Ofuka-cho, Kita-ku, Osaka, 530-8552 Japan

**Keywords:** Acquired blepharoptosis, Alpha-adrenergic agonist, Oxymetazoline hydrochloride 0.1%

## Abstract

**Purpose:**

To assess the 6-month efficacy and safety of oxymetazoline hydrochloride ophthalmic solution 0.1% (OMZ 0.1%) versus placebo for acquired blepharoptosis.

**Study design:**

A phase 3, randomized, double-masked, parallel-group, multicenter study conducted in Japan.

**Methods:**

Patients were randomized 1:1:1 to OMZ 0.1%, once daily (QD) or twice daily (BID), or placebo. Patients received OMZ 0.1% for 6 months. Patients in the placebo group were randomized after 3 months (Treatment Period 1) to OMZ 0.1%, either QD or BID, for the remaining 3 months (Treatment Period 2). The primary efficacy endpoint was the marginal reflex distance-1 (MRD-1) change from baseline to Day 14 (2 hours after the morning drop) with OMZ 0.1% QD or BID versus placebo. Adverse events and adverse drug reactions (ADRs) were recorded.

**Results:**

Overall, baseline MRD-1 (standard deviation) of 336 patients analyzed (n=112 per group) was 1.31 (0.83) mm (Day 0). MRD-1 change from baseline to Day 14 (2 hours after the morning drop) was 1.09 (0.07), 0.93 (0.07), and 0.50 (0.07) mm, with OMZ 0.1% QD, BID, and placebo, respectively. There was a statistically significant difference in the least squares mean (standard error) MRD-1 change versus placebo with OMZ 0.1% QD (0.59 [0.10] mm; 95% CI 0.38, 0.79) and OMZ 0.1% BID (0.43 [0.10] mm; 95% CI 0.23, 0.64) (both *p*<0.05). All ADRs related to the study drug were mild.

**Conclusion:**

Fourteen days of OMZ 0.1% treatment significantly increased MRD-1 versus placebo. There was no loss of effect after 6 months of treatment and no significant safety concerns.

**Supplementary Information:**

The online version contains supplementary material available at 10.1007/s10384-026-01340-5.

## Introduction

Blepharoptosis is a common eyelid condition characterized by drooping of the upper eyelid, which can be either acquired or congenital. Blepharoptosis is estimated to affect 4.7–13.5% of adults, and prevalence increases with age [1]. In addition, blepharoptosis is reported to be associated with long-term wear of hard contact lenses [2, 3]. Even in milder cases, blepharoptosis can lead to functional visual impairment and, in turn, limit social interactions that can result in a decreased quality of life for patients [2, 4].

Treatment options for blepharoptosis are mostly surgical and aim to target the dysfunctional upper eyelid retractor muscles [5]. However, surgery, which is the main treatment approach in Japan, can be associated with risks such as asymmetry, bleeding, and infection [4, 6]. The addition of pharmacological interventions for acquired blepharoptosis could widen the available treatment options, particularly for patients who are not comfortable with surgery [1]. Off-label use of alpha-adrenergic agents, such as phenylephrine, apraclonidine, and naphazoline, developed for other ophthalmic applications, is reported in blepharoptosis, including myasthenia gravis [1, 7–9]. However, these reports are limited in scope [1], and further evaluation through clinical trials is required.

As there are currently no approved pharmacological treatments for blepharoptosis in Japan [6], there remains an unmet need for a non-surgical, pharmacological treatment for patients with mild-to-moderate blepharoptosis, particularly when surgery is not suitable.

Oxymetazoline hydrochloride ophthalmic solution 0.1% (OMZ 0.1%) is an alpha-adrenergic agonist that stimulates the Müller muscle of the upper eyelid, causing muscle contraction and lifting of the eyelid [10]. Two confirmatory, phase 3 studies conducted in the United States reported the positive efficacy and safety of OMZ 0.1% for up to Day 42 of administration [4]. The mean (standard deviation [SD]) change from baseline in the number of points seen in the superior visual field as measured by the Leicester Peripheral Field Test (LPFT), a test to detect visual impairment due to blepharoptosis, was significantly greater in participants receiving OMZ 0.1% when compared with a vehicle administration in both studies at Day 14 (7.1 [5.9] vs. 2.4 [5.5]; mean difference *p*<0.001) [4].

In a pooled analysis of the above phase 3 studies, once daily (QD) OMZ 0.1% was shown to significantly and rapidly improve marginal reflex distance-1 (MRD-1) 5 minutes post-administration, sustained up to Day 42, when compared with placebo (*p*<0.05) [2]. In terms of safety, a pooled analysis of four randomized, double‑masked clinical trials showed that OMZ 0.1%, administered QD, was well tolerated in patients with acquired blepharoptosis when used for up to 84 days [10].

The efficacy and safety of OMZ 0.1% has yet to be reported in a Japanese patient population. The present study aimed to assess the 6-month efficacy and safety of OMZ 0.1% versus placebo in Japanese patients with acquired blepharoptosis.

## Patients and methods

### Study design

This phase 3, randomized, double-masked, parallel-group, multicenter study in patients with acquired blepharoptosis was designed to investigate the superiority of OMZ 0.1% versus placebo in the MRD-1 change from baseline at Day 14 up to a maximum of 6 months. This study was registered at the Japan Registry of Clinical Trials (jRCT2031220394) and was conducted at 38 institutions in Japan from October 11, 2022, to February 7, 2024. The institutional review board at each study site approved the study protocol, and written informed consent was obtained before the study began. The names of the institutional review boards for each site are listed in Supplementary Appendix 1. The study was conducted in accordance with Good Clinical Practice and the ethical principles of the Declaration of Helsinki.

**Fig. 1:**
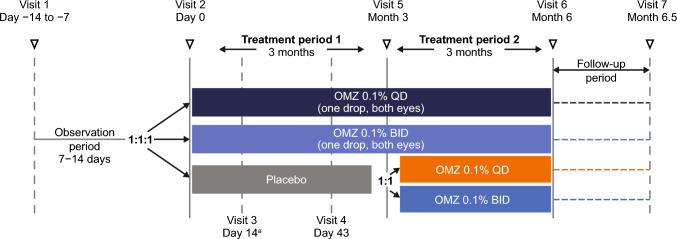
OMZ 0.1% confirmatory study design. a Primary efficacy endpoint evaluation at 2 hours after the morning drop on Day 14, key secondary efficacy evaluation at 15 minutes after the evening drop on Day 14. BID, twice daily; OMZ 0.1%, oxymetazoline hydrochloride ophthalmic solution 0.1%; QD, once daily

Eligibility was assessed during the observation period from Day –14 to –7 (Fig. 1). All eligible patients entered Treatment period 1 (3 months) consecutively; they were randomized 1:1:1 (using the Randomization and Trial Supply Management system) to either OMZ 0.1% (administered QD or twice daily [BID]), or placebo (administered BID) for the 3 months. OMZ 0.1% or placebo eye drops were administered in the morning (9.30 a.m. ±1 hour) and evening (5.30 p.m. ±1 hour) each day from Visit 2 (Day 0). Evening drops were administered 8 hours (±1 hour) after the morning drops. To maintain double-masking, patients in the OMZ 0.1% QD group received the study drug in the morning and placebo in the evening. Therefore, a placebo QD group was not included in the study design.

To check whether the effects of oxymetazoline decreased with continuous use, attenuation of efficacy was evaluated at Month 3. If a decrease in responsiveness was observed, a 14-day (±3 days) washout period was set, during which OMZ 0.1% treatment was suspended. The criteria for treatment suspension were based on changes in MRD-1; patients whose MRD‑1 value had decreased by 1.0 mm or more from Day 14 were selected for the washout period.

For all those deemed eligible for Treatment period 2, treatment continued for an additional 3 months, for a total of 6 months (Fig. 1). Those patients who received the placebo during Treatment period 1 were randomized 1:1 at Month 3 to OMZ 0.1% either QD or BID for the remaining 3 months of the study, if eligible. Eligible patients who had received OMZ 0.1% in Treatment period 1 were continued on the same dosing (QD or BID) through Treatment Period 2. After completing 6 months of treatment, patients returned for a follow-up visit 14 days later, and MRD-1 was measured. Assessment of rebound following treatment cessation, defined as worsening blepharoptosis, was carried out by the investigator. Presence of rebound was determined by comparing the MRD-1 value recorded at follow-up with the baseline MRD-1 value. The baseline MRD-1 value was defined as the measurement recorded immediately before treatment initiation on Day 0.

MRD-1 values were collected at 5±2 minutes, 15±2 minutes, and 2 hours±30 minutes after the morning treatment, and 5±2 minutes and 15±2 minutes after the evening treatment at each visit (Visit 2 [Day 0], Visit 3 [Day 14], Visit 4 [Day 43], Visit 5 [Month 3], and Visit 6 [Month 6]). Further information about MRD-1 assessments, including additional timepoints for MRD-1 evaluation, are presented in Supplementary Appendix 1.

The study eye for efficacy evaluation was defined as the eye that had the lower MRD-1 value at screening (indicating greater severity). If both eyes had equal MRD-1 values (including both considered as MRD-1 = 0), the right eye was designated as the study eye.

### Participants and procedure

Key inclusion criteria at the time of written consent included male and female patients, aged ≥18 to <75 years, with a diagnosis of acquired blepharoptosis in ≥1 eye. Patients must have had an MRD-1 value of ≤2 mm at the start of the observation period and a corrected visual acuity ≤1.00 logarithm of the Minimum Angle of Resolution.

Key exclusion criteria were the presence of any of the following conditions: pseudo- or congenital blepharoptosis, Horner syndrome, Marcus Gunn jaw-winking syndrome, myasthenia gravis, and mechanical eyelid blepharoptosis. Additional exclusion criteria are listed in Supplementary Appendix 1.

### Outcomes

The primary efficacy endpoint was the MRD-1 change from baseline to Day 14 (2 hours after the morning drop on that day) with OMZ 0.1% QD or BID versus placebo. The key secondary efficacy endpoint was the MRD-1 change from baseline to Day 14 (15 minutes after the evening drop on that day) with OMZ 0.1% QD or BID versus placebo. Other secondary endpoints included MRD-1 change from baseline to each timepoint up to Month 6 with OMZ 0.1% QD or BID versus placebo. Quality of life (QoL) was assessed at the start of the observation period and before the morning drop on the following visits: Day 14, Month 3, Month 6, and at the time of discontinuation. QoL was assessed using the United States National Eye Institute Visual Function Questionnaire-25 (VFQ-25).

To evaluate the consistency in efficacy of OMZ 0.1% in different patient populations, a subgroup analysis of the primary endpoint was performed. Subgroups were defined by sex (male or female), age (≤59 or ≥60 years), and baseline MRD-1 value (<1, 1 to <2, or ≥2 mm).

The safety endpoint was the tolerability of OMZ 0.1% administered QD and BID. Safety evaluations included adverse events (AEs), and adverse drug reactions (ADRs), defined as AEs that were causally related to the study drug, as determined by the investigator. The following safety variables were recorded at the visits listed or at the time of treatment discontinuation: intraocular pressure (IOP) and pupil diameter were measured in both eyes at Visits 1, 5, and 6. In addition, slit-lamp examinations were performed at all visits and at the time of treatment discontinuation. Further details are provided in Supplementary Appendix 1.

### Statistical analyses

It was determined through a two-sided t-test (α = 0.05) that 101 patients would be required in each treatment group to provide a statistical power of 90%. Therefore, the target sample size was 324 participants, 108 participants in each of the three groups in Treatment period 1, and 162 in both groups in Treatment period 2, after assuming a dropout rate of 5% by Day 14.

The planned sample size was based on observing an assumed difference in mean MRD-1 change from baseline at Day 14 (2 hours after first treatment) of 0.5 mm between the OMZ 0.1% QD and BID groups and placebo, with an SD of 1.0 mm. The primary and key secondary efficacy endpoints were assessed using a mixed-effect model for repeated measures (MMRM) analysis, which included treatment, visit, treatment‑visit interaction, and the interaction between MRD-1 value at baseline and the evaluation timepoint as the fixed effects; MRD-1 at the start of treatment as the covariate; and patients as the random effects. The Truncated Hochberg method using two-stage gatekeeping was performed for the primary and key secondary endpoints, to control for the probability of type I errors in the comparisons between OMZ 0.1% QD, BID, and placebo. Unless otherwise specified, the significance level for statistical tests was set at 5% (two-sided), and the confidence level for interval estimation was set at 95% (two-sided).

The intention-to-treat population included all patients randomized to a treatment group. Efficacy analyses were conducted on the full analysis set, defined as randomized patients who met the eligibility criteria, received ≥1 dose of their allocated medication, and from whom MRD-1 data were collected. Enrolled patients who received ≥1 dose of their allocated medication and who provided any safety data were included in the safety analysis set population.

## Results

### Patient disposition

**Fig. 2:**
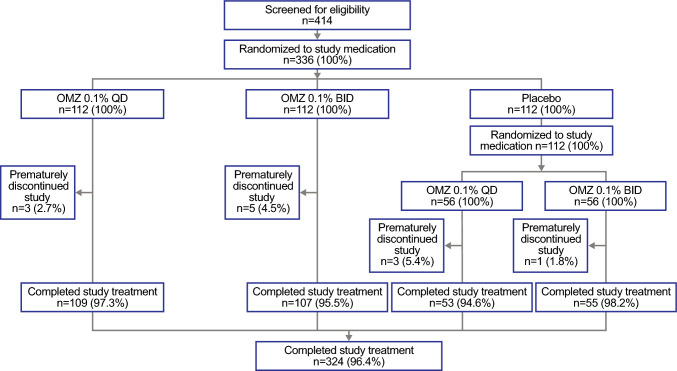
Patient disposition. BID, twice daily; OMZ 0.1%, oxymetazoline hydrochloride 0.1%; QD, once daily

A total of 336 patients were randomized to OMZ 0.1% QD (n=112), OMZ 0.1% BID (n = 112), or placebo (n = 112) for Treatment period 1. Patient disposition is outlined in Fig. 2. Patients receiving placebo in Treatment period 1 were then randomized 1:1 to OMZ 0.1% QD (n = 56) or BID (n = 56) for 3 months (Treatment period 2).

Demographics and baseline characteristics are summarized in Table 1. Baseline characteristics were balanced between treatment groups. Overall, median (range) age was 60.0 (18, 74), and 85.7% of patients were female. Mean (SD) MRD-1 at baseline was 1.31 (0.83) mm.

**Table 1:** Baseline characteristics (intention-to-treat population)

Characteristic	OMZ % QD (n=112)	OMZ 0.1% BID (n=112)	Placebo (n=112)	Overall (N=336)
Age, median (range), years	59.0 (18, 74)	59.5 (32, 74)	61.0 (29, 74)	60.0 (18, 74)
≤49 years, n (%)	16 (14.3)	16 (14.3)	15 (13.4)	47 (14.0)
50–59 years, n (%)	41 (36.6)	40 (35.7)	37 (33.0)	118 (35.1)
60–69 years, n (%)	28 (25.0)	39 (34.8)	37 (33.0)	104 (31.0)
≥70 years, n (%)	27 (24.1)	17 (15.2)	23 (20.5)	67 (19.9)
Female, n (%)	97 (86.6)	92 (82.1)	99 (88.4)	288 (85.7)
Race, *n* (%)				
Japanese	112 (100.0)	111 (99.1)	112 (100.0)	335 (99.7)
American Indian or Alaska Native	0	1 (0.9)	0	1 (0.3)
MRD-1^a^, mean (SD), mm	1.25 (0.85)	1.38 (0.80)	1.31 (0.85)	1.31 (0.83)
<1 mm, n (%)	44 (39.3)	40 (35.7)	42 (37.5)	126 (37.5)
≥1 to <2 mm, n (%)	44 (39.3)	47 (42.0)	51 (45.5)	142 (42.3)
≥2 mm, n (%)	24 (21.4)	25 (22.3)	19 (17.0)	68 (20.2)
Visual acuity, mean (SD), logMAR	–0.03 (0.10)	–0.01 (0.16)	–0.03 (0.10)	–0.02 (0.12)

BID, twice daily; logMAR, logarithm of the Minimum Angle of Resolution; MRD-1, marginal reflex distance-1; OMZ 0.1%, oxymetazoline hydrochloride 0.1%; QD, once daily

^a^Reported MRD-1 values were recorded immediately before treatment initiation. The <2 mm cut-off was only applied to MRD-1 values observed during the observation period. Patients with an MRD-1 value of ≥2 mm shown here were included as a result of MRD-1 fluctuations between the observation period and start of treatment; these patients would have had an MRD-1 of <2 mm during the observation period in order to be eligible for the study

### Efficacy

For the primary efficacy endpoint, the adjusted (least squares [LS]) mean (standard error [SE]) for the MMRM analysis of the MRD-1 change from baseline (Day 0) to Day 14 (2 hours after the morning drop) was 1.09 (0.07) mm with OMZ 0.1% QD, 0.93 (0.07) mm with OMZ 0.1% BID, and 0.50 ± 0.07 mm with placebo (Table 2).

**Table 2:** Primary and key secondary efficacy endpoints

Efficacy endpoint (timepoint)	Treatment group (n)	LS mean (SE) MRD-1 (mm)	LS mean difference from placebo (mm)	95% CI of difference	*p*-value^a^
Primary (MRD-1 change from baseline to Day 14, 2 hours after the morning drop)	OMZ 0.1% QD (n=112)	1.09 (0.07)	0.59 (0.10)	0.38, 0.79	0.0000
	OMZ 0.1% BID (n=112)	0.93 (0.07)	0.43 (0.10)	0.23, 0.64	0.0000
	Placebo (n=112)	0.50 (0.07)	NA	NA	NA
Key secondary (MRD-1 change from baseline to Day 14, 15 minutes after the evening drop)	OMZ 0.1% QD (n=112)	0.90 (0.07)	0.36 (0.10)	0.16, 0.56	0.0005^b^
	OMZ 0.1% BID (n=112)	0.79 (0.07)	0.25 (0.10)	0.05, 0.45	0.0142
	Placebo (n=112)	0.54 (0.07)	NA	NA	NA

BID; twice daily; LS, least squares; MRD-1, marginal reflex distance-1; NA, not available; OMZ 0.1%, oxymetazoline hydrochloride 0.1%; QD, once daily; SE, standard error

A two-stage gatekeeping procedure using Truncated Hochberg method (truncated parameter=0.7) was performed to control for multiplicity of the primary endpoint; a two-sided significance level of 0.025 and 0.0425 was set for the primary endpoint evaluating OMZ 0.1% QD and BID, respectively, versus placebo. The significance level for the secondary endpoint was set at 0.05

^a^Mixed-effect model for repeated measures analysis of the variance between OMZ 0.1% QD or BID versus placebo. Treatment, visit, treatment-visit interaction, and the interaction between MRD-1 value at baseline and the evaluation timepoint were used as the fixed effects; MRD-1 at the start of treatment was used as the covariate; and patients were used as the random effects

^b^Nominal *p*-value; the key secondary efficacy endpoint was powered for the OMZ 0.1% BID group only

A statistically significant difference in the LS mean MRD-1 change from baseline at Day 14 (2 hours after the morning drop) was observed between both OMZ 0.1% dosing groups and placebo. The difference in the LS mean (SE) change from baseline in MRD-1 versus placebo was 0.59 (0.10) mm (95% CI 0.38, 0.79) with OMZ 0.1% QD and 0.43 (0.10) mm (95% CI 0.23, 0.64) with OMZ 0.1% BID. The *p-*values obtained in the MMRM analysis for both OMZ 0.1% dosing groups were *p*=0.0000, smaller than the 0.0425 significance level obtained using the Truncated Hochberg method, based on the two-stage gatekeeping procedure. Therefore, the superiority of the change from baseline in MRD-1 to Day 14 (2 hours after the morning drop) was verified in both the OMZ 0.1% QD and BID groups, versus placebo.

The adjusted LS mean (SE) for the MMRM analysis of the MRD-1 change from baseline to Day 14 (15 minutes after the evening drop), the key secondary efficacy endpoint, was 0.90 (0.07) mm with OMZ 0.1% QD, 0.79 (0.07) mm with OMZ 0.1% BID versus 0.54 (0.07) mm with placebo (Table 2). The difference in the LS mean (SE) MRD-1 change from baseline versus placebo was 0.36 (0.10) mm (95% CI 0.16, 0.56) in the OMZ 0.1% QD group and 0.25 (0.10) mm (95% CI 0.05, 0.45) in the OMZ 0.1% BID group. The *p-*value obtained in the MMRM analysis for the BID group, 0.0142, was smaller than the pre-determined significance level of 0.05 that was obtained via the Truncated Hochberg method. Therefore, a statistically significant difference was observed between OMZ 0.1% BID and placebo (*p<*0.05). A similar trend was also observed with the OMZ 0.1% QD group (nominal *p*=0.0005).

**Fig. 3:**
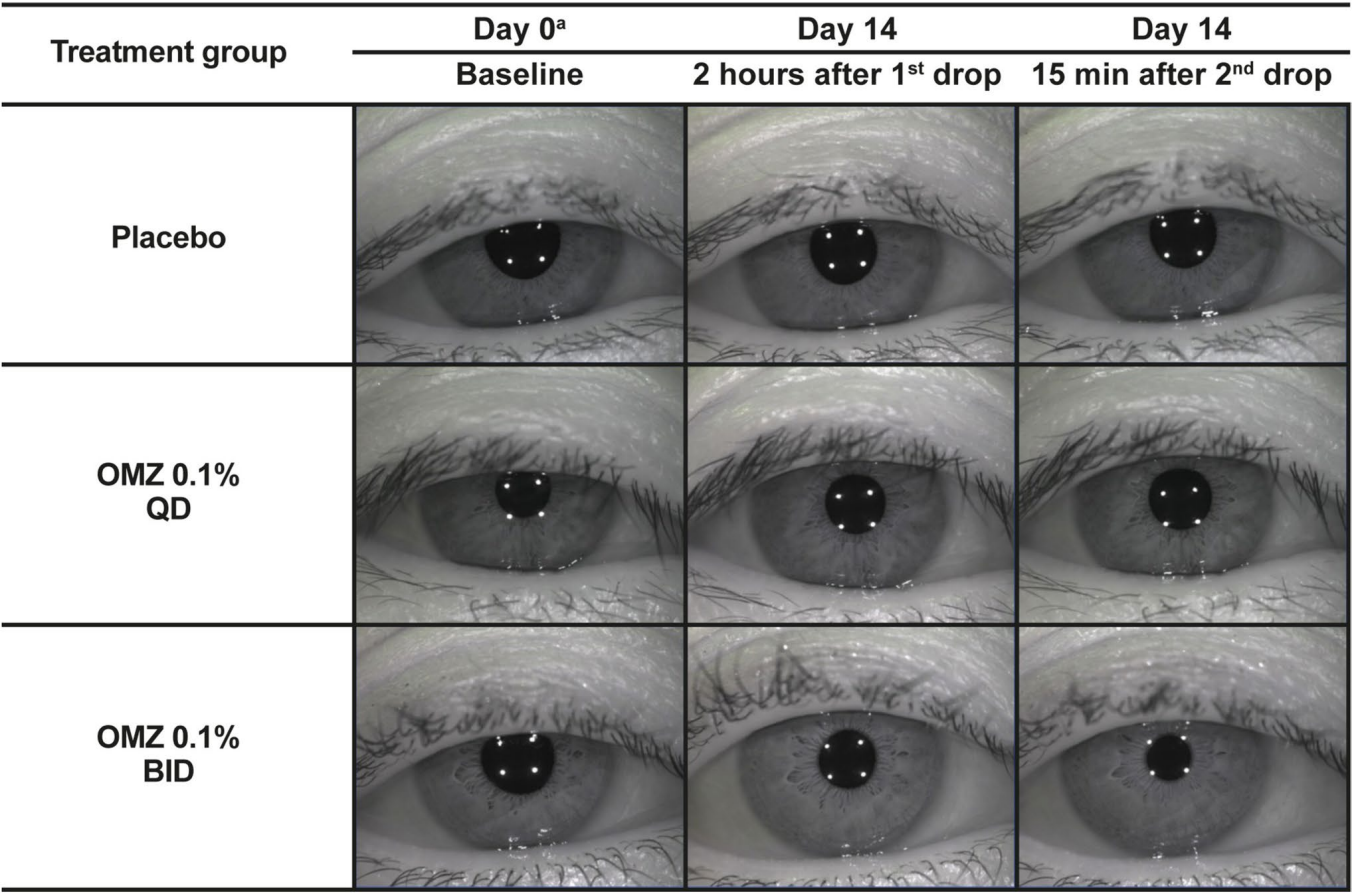
Photographs showing representative patient examples of changes in marginal reflex distance-1 (change in upper eyelid position) after OMZ 0.1% treatment versus placebo. ^a^Treatment initiation. BID, twice daily; OMZ 0.1%, oxymetazoline hydrochloride 0.1%; QD, once daily

Photographs showing representative patient examples of the observed changes in upper eyelid position, reflective of MRD-1 changes, from baseline to Day 14 in patients treated with OMZ 0.1% versus placebo are presented in Fig. 3.

**Fig. 4:**
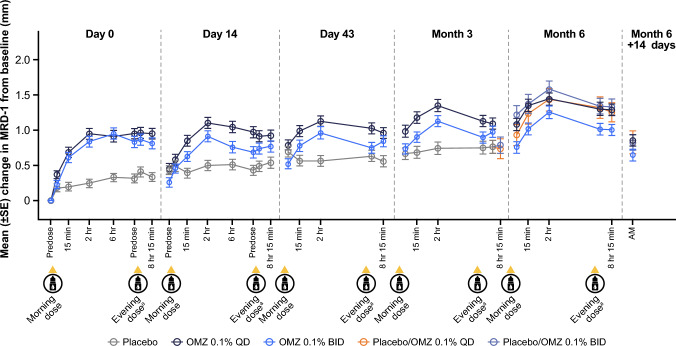
Change from baseline in MRD-1 at each timepoint and visit to Month 6. ^a^Patients in the QD group received placebo in the evening, for masking purposes. BID, twice daily; MRD-1, marginal reflex distance-1; OMZ 0.1%, oxymetazoline hydrochloride 0.1%; QD, once daily

MRD-1 changes from baseline at each evaluation timepoint, a secondary efficacy endpoint, demonstrated that the effects of OMZ 0.1% after the first administration were sustained for several hours. The 95% CI of the difference between the OMZ 0.1% QD group and placebo did not cross zero, starting at 15 minutes after treatment and continuing up to 8 hours and 15 minutes post-administration (Table S1). In addition, continuous improvements in MRD-1 were observed in both OMZ 0.1% dosing groups until Month 6 (Fig. 4).

Consistent with the primary efficacy endpoint, subgroup analysis by baseline MRD-1 value (<1, 1 to <2, or ≥2 mm), age (≤59 or ≥60 years), and sex (male or female) demonstrate a greater MRD-1 change from baseline to Day 14 (2 hours after the morning drop) with OMZ 0.1% QD and BID versus placebo across all subgroups (Fig. S1).

At Month 3, there was no attenuation of efficacy in either OMZ 0.1% dosing groups, and all patients continued treatment without entering the washout period. During the follow-up period (Month 6+14 days), there were two cases (0.9%) of rebound, as judged by the investigator, following treatment cessation. Both cases occurred in the OMZ 0.1% QD group.

No significant improvements in QoL (as assessed by VFQ-25) from the start of treatment were observed with either OMZ 0.1% dosing groups versus placebo at any timepoint.

### Safety

A total of 336 patients were included in the safety analysis set for Treatment period 1 (n=112; all groups) and 329 patients for Treatment period 2 (OMZ 0.1% QD, n=110; OMZ 0.1% BID, n=109; placebo/OMZ 0.1% QD and placebo/OMZ 0.1% BID, n=55 in each group).

During Treatment period 1, ADRs were reported in 0.9% (n = 1/112) of patients receiving OMZ 0.1% QD, and 4.5% of patients receiving OMZ 0.1% BID or placebo (n=5/112 in each group) (Table 3). During Treatment period 2, ADRs were reported in zero patients receiving OMZ 0.1% QD and 4.6% (n=5/109) of patients receiving OMZ 0.1% BID. In patients who were switched from the placebo to either OMZ 0.1% QD or BID, ADRs occurred in one patient out of 55 (1.8%) from each group (Table 4). For a full list of AEs occurring at a rate of ≥1% in Treatment periods 1 and 2, see Tables S2 and S3.

**Table 3:** Summary of ADRs: Treatment period 1

Patients, n (%)	OMZ 0.1% QD (n=112)	OMZ 0.1% BID (n=112)	Placebo (n=112)
Any ADR^a^	1 (0.9)	5 (4.5)	5 (4.5)
*Specific ADRs (MedDRA preferred term) occurring at a rate of ≥1%*			
Punctate keratitis	0 (0.0)	2 (1.8)	1 (0.9)
Vision blurred	0 (0.0)	1 (0.9)	2 (1.8)
Conjunctival hyperemia	0 (0.0)	1 (0.9)	0 (0.0)

ADR, adverse drug reaction; BID, twice daily; MedDRA, Medical Dictionary for Regulatory Activities; OMZ 0.1%, oxymetazoline hydrochloride 0.1%; QD, once daily

^a^ADRs were adverse events judged by the investigator to be causally related to the study drug

**Table 4:** Summary of ADRs: Treatment period 2

Patients, n (%)	OMZ 0.1% QD (n=110)	OMZ 0.1% BID (n=109)	Placebo/OMZ 0.1% QD (n=55)	Placebo/OMZ 0.1% BID (n=55)
Any ADR^a^	0 (0.0)	5 (4.6)	1 (1.8)	1 (1.8)
*Specific ADRs (MedDRA preferred term) occurring at a rate of ≥1%*				
Punctate keratitis	0 (0.0)	1 (0.9)	0 (0.0)	1 (1.8)
Vision blurred	0 (0.0)	1 (0.9)	1 (1.8)	0 (0.0)
Conjunctival hyperemia	0 (0.0)	2 (1.8)	0 (0.0)	0 (0.0)

ADR, adverse drug reaction; BID, twice daily; MedDRA, Medical Dictionary for Regulatory Activities; OMZ 0.1%, oxymetazoline hydrochloride 0.1%; QD, once daily

^a^ADRs were adverse events judged by the investigator to be causally related to the study drug

A total of nine moderate AEs were reported throughout the study across Treatment periods 1 and 2, all of which were determined to be unrelated to the study drug. Four moderate AEs were reported during Treatment period 1 (OMZ 0.1% BID, n=3; placebo, n=1) and five were reported during Treatment Period 2 (OMZ 0.1% QD, n=1; OMZ 0.1% BID, n=1; placebo/OMZ 0.1% BID, n=1; placebo/OMZ 0.1% QD, n=2). All other events were assessed as mild, with no severe AEs reported.

Serious AEs (SAEs) occurred in two patients receiving OMZ 0.1% BID in Treatment period 1 and one patient who was switched from placebo to OMZ 0.1% QD during Treatment period 2. No SAEs were deemed to be related to treatment. For further details about the reported SAEs, see Supplementary Appendix 2. As an AE of interest, only three cases of conjunctival hyperemia were reported in two patients who received OMZ 0.1% BID. One case of conjunctival hyperemia began in Treatment period 1 during Month 3 and lasted 90 days. The two other cases began in Treatment Period 2, during Months 4 and 5 for a duration of 20 and 91 days, respectively. All of these events were mild and resolved after treatment cessation. No deaths or AEs leading to study discontinuation in either treatment period were reported.

The changes in pupillary diameter and IOP from baseline to Month 6 were minimal across all groups. Further data are available in Supplementary Appendix 2.

## Discussion

This study supports the efficacy and safety of OMZ 0.1% in both QD and BID dosing in Japanese patients with acquired blepharoptosis who were treated for up to 6 months. The primary efficacy endpoint (superiority of OMZ 0.1% BID or QD vs. placebo in MRD-1 change from baseline to Day 14 [2 hours after the morning drop on that day]) was met (both *p*<0.05). The key secondary endpoint (superiority in MRD-1 change to Day 14 [15 minutes after the evening drop on that day]) was also met in the OMZ 0.1% BID group versus placebo (*p*<0.05). A similar trend was observed in the OMZ 0.1% QD group (nominal *p*=0.0005), demonstrating that MRD-1 improvements persisted for up to 8 hours 15 minutes after the morning drop. This efficacy trend continued with MRD-1 improvement reported throughout Treatment period 2, up to Month 6, in both OMZ 0.1% dosing groups and in patients who switched from placebo to OMZ 0.1%.

Across all subgroups evaluated, the treatment effect was mostly consistent with that observed in the overall study population. Patients with a baseline MRD-1 ≥2 mm exhibited a lower effect than other MRD-1 subgroups. This is likely because those with milder blepharoptosis have a smaller improvement range than patients with more severe disease. This subgroup also comprised patients whose MRD-1 was <2 mm at screening but had increased to ≥2 mm by baseline, suggesting the possibility of regression or selection bias. Therefore, these subgroup findings should be interpreted with caution.

Previous phase 3 studies have evaluated both MRD-1 and the number of points seen in the superior visual field (via LPFT) data to evaluate the benefit of OMZ 0.1% in blepharoptosis [2, 4]. In this study, MRD-1 was used to measure blepharoptosis improvement, based on a report by the American Academy of Ophthalmology. MRD-1 is an internationally recognized measure widely used to assess blepharoptosis [11], and recommendations suggest that it is the most predictive indicator of visual field defects in blepharoptosis [5]. Based on the above, MRD-1 was selected as the primary endpoint of the current trial.

The MRD-1 improvement after 14 days of OMZ 0.1% treatment observed in this study was similar to the efficacy reported in a previous study of a US population [2], where mean (SD) MRD-1 change from baseline to Day 14 (5 minutes post-administration) was 0.77 (0.85) mm with OMZ 0.1% QD versus 0.42 (0.78) mm with the vehicle (*p*=0.015). Previous studies demonstrate a strong correlation between MRD-1 and super visual field, with one report indicating that an MRD-1 value of ≤2 mm corresponds with a 12–15 degree loss in superior visual field [5]. A prospective case series of 52 patients demonstrates that an MRD-1 increase of 0.8–3.1 mm following blepharoptosis surgery was associated with a mean improvement of 13.6 degrees in the superior visual field [12]. Similarly, in the US-based phase 3 trials, OMZ 0.1% treatment was associated with improvements in the superior visual field alongside MRD-1 elevation [4]. Although visual field measurements were not performed in the present study, the observed mean MRD-1 improvement of approximately 1 mm suggests a clinically meaningful change that likely translates into superior visual field enhancement.

It has been reported that prolonged use of alpha-adrenergic receptor agonists can lead to the loss of effectiveness via the downregulation of alpha-adrenoceptors and blepharoptosis rebound after treatment cessation [13–15]. In this study, no attenuation of efficacy was observed after 3 months of continuous OMZ 0.1% treatment, with continuous improvements in MRD-1 up to Month 6. Further, at Month 6+14 days, only two (0.9%) patients reported blepharoptosis rebound, defined by worsening MRD-1, after cessation of OMZ 0.1% treatment.

Among those patients receiving OMZ 0.1% in this study, AE rates up to Month 6 (24.8–30.9%) were similar to those reported up to Day 42 (31.0%) in two randomized, double-masked, phase 3 clinical trials conducted in the US, also in patients with acquired blepharoptosis (RVL-1201-201 and -202) [4]. OMZ 0.1% targets alpha-adrenergic receptors, which are often in abundance in the smooth muscle in the iris and blood vessels in the conjunctiva; this may provide an explanation for the reports of ocular AEs such as dry eye, punctate keratitis, and conjunctival hyperemia with OMZ 0.1% use [10, 16]. However, no cases of dry eye were determined to be related to the study drug in this study.

In a pooled analysis of the safety of OMZ 0.1% reported in the RVL‑1201‑001, -201, -202, and -203 studies, dry eye judged to be treatment-related (i.e. a treatment-emergent AE; TEAE), was reported by 1.6% of patients receiving OMZ 0.1% over 42 days [10]. A history of dry eye was reported in 43.8% of patients from the RVL‑1201 studies, suggesting a susceptibility to corneal irritation in this patient population [10].

Furthermore, reactive hyperemia was another AE of interest, due to the specific mechanism of action of OMZ 0.1% as an alpha-adrenergic agonist [13]. Conjunctival hyperemia was reported in only one (0.4%) patient receiving OMZ 0.1% in Treatment period 1, and two (0.6%) patients during Treatment period 2. As all cases of conjunctival hyperemia were reported during study dosing and ceased once dosing ended, they were judged not to be reactive events. Conjunctival hyperemia was reported as a TEAE in 2.9% of participants receiving OMZ 0.1% at Day 42 in the pooled safety analysis of the RVL-1201 studies [10]. The most commonly reported AE in the previous US studies, punctate keratitis (reported in 5.4% of patients) [2], was observed in only 0.9–2.7% of patients receiving OMZ 0.1% in this study. The reason why there were relatively few AEs of dry eye and conjunctival hyperemia in this trial compared with the US trial is unclear, but it is possible that the use of dry eye medications was permitted in cases of dry eye, which may have had an impact.

A strength of this study was that most participants (96.4%) completed the study treatment. Additionally, continued monitoring of MRD-1 values up to Month 6 allowed longer-term trends in efficacy to be evaluated and the level of blepharoptosis rebound to be determined.

This study had some limitations. Patients with acquired blepharoptosis secondary to myasthenia gravis or other forms of blepharoptosis are excluded, and their efficacy has not been studied. Most participants were female (85.7%); however, there was no imbalance between groups that could have impacted the efficacy assessments for any of the background factors. This study did not demonstrate any significant changes in VFQ-25, and it may not have fully captured QoL related to the specific symptoms of blepharoptosis. Moreover, the impact on other dimensions of QoL, including overall well-being and psychological aspects, remains uncertain. Further research is warranted to determine how blepharoptosis improvement with OMZ 0.1% translates into QoL changes. Additionally, functional outcomes, such as visual field changes, were not assessed. Finally, this study was not powered to compare OMZ 0.1% QD with BID, so no conclusions should be drawn about the relative efficacy of the different dosing frequencies.

Collectively, these results show that in Japanese patients with acquired blepharoptosis enrolled in this study, OMZ 0.1% QD and BID significantly increased MRD-1 versus placebo for 14 days. The reported MRD-1 improvements with OMZ 0.1% persisted for over 8 hours after initial administration. Furthermore, continuous MRD-1 improvements were observed during the 6 months of treatment for both OMZ 0.1% dosing groups, with no attenuation of efficacy observed and only a small number of patients experiencing blepharoptosis rebound following treatment cessation. There were no unexpected safety concerns identified, and the safety profile reported here is similar to that seen in other clinical studies [2, 4, 10]. Our findings suggest that OMZ 0.1% could be considered as an initial treatment option for Japanese patients. OMZ 0.1% may help address the unmet need in Japan for blepharoptosis care for patients who may not be eligible for or prefer to avoid blepharoptosis surgery.

## Supplementary Information

Below is the link to the electronic supplementary material.

Supplementary file1 (PDF 202 KB)

Supplementary file2 (PDF 119 KB)

Supplementary file3 (PDF 173 KB)

Supplementary file4 (PDF 104 KB)

Supplementary file5 (PDF 138 KB)

Supplementary file6 (PDF 157 KB)

Supplementary file7 (PDF 626 KB)

## References

[1] Bacharach JA-O, Lee WW, Harrison AR, Freddo TF. A review of acquired blepharoptosis: prevalence, diagnosis, and current treatment options. Eye (Lond). 2021;35:2468–81. 33927356 10.1038/s41433-021-01547-5 PMC8376882

[2] Bacharach J, Wirta DL, Smyth-Medina R, Korenfeld MA-O, Kannarr SR, Foster SA-O, et al. Rapid and sustained eyelid elevation in acquired blepharoptosis with oxymetazoline 0.1%: randomized Phase 3 trial results. Clin Ophthalmol. 2021;14:2743–51. 10.2147/OPTH.S306155 PMC8240850 34211263

[3] Watanabe A, Imai K, Kinoshita S. Impact of high myopia and duration of hard contact lens wear on the progression of ptosis. Jpn J Ophthalmol. 2013;57:206–10. 23229097 10.1007/s10384-012-0222-8

[4] Slonim CB, Foster S, Jaros M, Kannarr SR, Korenfeld MS, Smyth-Medina R, et al. Association of oxymetazoline hydrochloride, 0.1%, solution administration with visual field in acquired ptosis: a pooled analysis of 2 randomized clinical trials. JAMA Ophthalmol. 2020;138:1168–75. 33001144 10.1001/jamaophthalmol.2020.3812 PMC7530825

[5] Cahill KV, Bradley EA, Meyer DR, Custer PL, Holck DE, Marcet MM, et al. Functional indications for upper eyelid ptosis and blepharoplasty surgery: a report by the American Academy of Ophthalmology. Ophthalmology. 2011;118:2510–7. 22019388 10.1016/j.ophtha.2011.09.029

[6] Kakizaki H. Blepharoptosis. Nihon Iji Shinpo. 2022;5108:46 (**(in Japanese)**).

[7] Agha M, Ismail H, Sawaya R, Salameh J. Efficacy of apraclonidine eye drops in treating ptosis secondary to myasthenia gravis: a pilot clinical trial. Muscle Nerve. 2023;68:206–10. 37259693 10.1002/mus.27851

[8] Nagane Y, Utsugisawa K, Suzuki S, Masuda M, Shimizu Y, Utsumi H, et al. Topical naphazoline in the treatment of myasthenic blepharoptosis. Muscle Nerve. 2011;44:41–4. 21491460 10.1002/mus.22002

[9] Wijemanne S, Vijayakumar D, Jankovic J. Apraclonidine in the treatment of ptosis. J Neurol Sci. 2017;376:129–32. 28431598 10.1016/j.jns.2017.03.025

[10] Wirta DL, Korenfeld MA-O, Foster SA-O, Smyth-Medina R, Bacharach J, Kannarr SR, et al. Safety of once-daily oxymetazoline HCl ophthalmic solution, 0.1% in patients with acquired blepharoptosis: results from four randomized, double-masked clinical trials. Clin Ophthalmol. 2021;15:4035–48. 34675472 10.2147/OPTH.S322326 PMC8517985

[11] Suga H, Takushima A. The aspect ratio of the palpebral fissure as a new blepharoptosis parameter. J Plast Surg Hand Surg. 2022;56:111–4. 34097563 10.1080/2000656X.2021.1934846

[12] Fuller ML, Briceño CA, Nelson CC, Bradley EA. Tangent screen perimetry in the evaluation of visual field defects associated with ptosis and dermatochalasis. PLoS ONE. 2017;12:e0174607. 28355310 10.1371/journal.pone.0174607 PMC5371337

[13] Hosten LO, Snyder C. Over-the-counter ocular decongestants in the United States –mechanisms of action and clinical utility for management of ocular redness. Clin Optom (Auckl). 2020;12:95–105. 32801982 10.2147/OPTO.S259398 PMC7399465

[14] Vaidyanathan S, Williamson P, Clearie K, Khan F, Lipworth B. Fluticasone reverses oxymetazoline-induced tachyphylaxis of response and rebound congestion. Am J Respir Crit Care Med. 2010;182:19–24. 20203244 10.1164/rccm.200911-1701OC

[15] Ishikawa H, Miller DD, Patil PN. Comparison of post-junctional alpha-adrenoceptors in iris dilator muscle of humans, and albino and pigmented rabbits. Naunyn Schmiedebergs Arch Pharmacol. 1996;354:765–72. 8971737 10.1007/BF00166903

[16] McAuliffe-Curtin D, Buckley C. Review of alpha adrenoceptor function in the eye. Eye. 1989;3:472–6. 2575042 10.1038/eye.1989.71

