# Supplementary material for: Six-month efficacy and safety of oxymetazoline hydrochloride 0.1% in Japanese patients with acquired blepharoptosis: a phase 3 study
Source: Jpn J Ophthalmol. 2026 Apr 10;70(4):820–9. doi: 10.1007/s10384-026-01340-5 (PMC13452870; doi:10.1007/s10384-026-01340-5)
Supplement: Supplementary file 1 [file 10384_2026_1340_MOESM1_ESM.pdf]

Supplementary Appendix 1 Additional methods

Institutional review boards (IRBs) involved in the study

- Medical Corporation Heishinkai OPHAC Hospital IRB
- Kobori Central Clinical Research Ethics Committee
- Keihin Chuo Clinic, Hisamitsu Clinic, and Shorin Hospital Joint Clinical Trial Review Board
- Shin-Akasaka Clinic Aoyama Institutional Review Board
- Institutional Review Board of Hayashi Eye Hospital
- Maebashi Hirosegawa Clinic Institutional Review Board
- Yamauchi Clinic Institutional Review Board

Additional exclusion criteria

Patients with a history of blepharoptosis surgery (excluding blepharoplasty ≥3 months prior), poor vision fixation in the frontal line of sight, or a history of angle-closure or narrow-angle glaucoma (excluding when peripheral iridotomy was performed ≥3 months prior to the start of the observation period) were excluded.

Marginal reflex distance-1 (MRD-1) assessments

MRD-1 values were determined using image data of the anterior segment of the eye generated by IDRA (INAMI Co., Ltd., Tokyo, Japan; manufactured by SBM Sistemi, Turin, Italy), including the pupils and upper eyelid margins. Calculations of MRD-1 values were conducted by an MRD-1 central measurement facility using collected image data and shared with the investigator. In addition to the MRD-1 evaluations conducted after treatment administration, additional data were collected at 6 hours ±30 minutes and 8 hours ±1 hour after the morning eyedrop application at Visits 2 [Day 0] and 3 [Day 14]. Baseline data and data from the follow-up period were collected at approximately 12.00 p.m. during the 7–14-day observation period before the start of the study (Visit 1) and the 14-day follow-up period after the study (Visit 7).

Additional assessments

Slit-lamp microscope examinations

The following examinations were carried out using a slit-lamp microscope at all visits and at the time of treatment discontinuation: the cornea was examined for edema and endothelial changes. In addition, fluorescein staining was conducted. The lens was evaluated for opacity, and the conjunctiva was evaluated for hyperemia and edema. The eyelid was evaluated for redness and edema. The limbal region was examined for swelling and Trantas dots. The anterior chamber was examined for cells, flare, and anterior and posterior synechia.

Concomitant medications

All concomitant medications (including treatment for dry eye) used during the clinical trial were documented
