# Supplementary material for: Six-month efficacy and safety of oxymetazoline hydrochloride 0.1% in Japanese patients with acquired blepharoptosis: a phase 3 study
Source: Jpn J Ophthalmol. 2026 Apr 10;70(4):820–9. doi: 10.1007/s10384-026-01340-5 (PMC13452870; doi:10.1007/s10384-026-01340-5)
Supplement: Supplementary file 2 [file 10384_2026_1340_MOESM2_ESM.pdf]

Supplementary Appendix 2 Additional safety results

Serious adverse events (SAEs)

The two SAEs reported in patients receiving oxymetazoline hydrochloride 0.1% (OMZ 0.1%) twice daily (BID) during Treatment Period 1 included one patient hospitalized with a radius fracture and one patient diagnosed with breast cancer. Neither SAE was deemed to be related to the study drug, but the patients were discontinued from the study. The one SAE reported in Treatment Period 2 was a diagnosis of schizophrenia in a patient who switched from placebo to OMZ 0.1% once daily (QD), which was determined as unrelated to the study drug. The patient was discontinued from the study.

Pupillary diameter and intraocular pressure (IOP) changes

The changes in pupillary diameter from baseline to Month 6 were minimal across all groups. Mean (SD) change from baseline to Month 6 (2 hours after first treatment) in pupil size were as follows: OMZ 0.1% QD, −0.0 (0.59) mm; OMZ 0.1% BID, –0.0 (0.58) mm; placebo to OMZ 0.1% QD, 0.1 (0.70) mm; and placebo to OMZ 0.1% BID, 0.0 (0.53) mm.

Similarly, the IOP change from baseline to Month 6 was minimal, and there were no increases in IOP in any group. Mean (SD) change from baseline to Month 6 (2 hours after first treatment) in IOP was as follows: OMZ 0.1% QD, –1.0 (2.41) mmHg; OMZ 0.1% BID, –1.1 (2.13) mmHg; placebo to OMZ 0.1% QD, –0.9 (2.00) mmHg; and placebo to OMZ 0.1% BID, –1.2 (2.03) mmHg. Changes in vital signs such as blood pressure and pulse rate showed little variation from baseline and were not clinically significant.
