# Supplementary material for: Six-month efficacy and safety of oxymetazoline hydrochloride 0.1% in Japanese patients with acquired blepharoptosis: a phase 3 study
Source: Jpn J Ophthalmol. 2026 Apr 10;70(4):820–9. doi: 10.1007/s10384-026-01340-5 (PMC13452870; doi:10.1007/s10384-026-01340-5)
Supplement: Supplementary file 3 [file 10384_2026_1340_MOESM3_ESM.pdf]

A study in Japan of eye drops to treat drooping upper eyelids

Plain language summary (938 words)

What did the study look at?

This study looked at how well eye drops containing 0.1% of a medicine called **oxymetazoline** improve a condition called acquired blepharoptosis in adults, and whether the eye drops cause any unwanted side effects when used once or twice a day.

What is acquired blepharoptosis?

Blepharoptosis is a condition in which the upper eyelid droops. This condition can affect one or both eyes and may be congenital, meaning it is present at birth, or acquired, meaning it develops later in life. Acquired blepharoptosis occurs because of weakening or damage to the muscles and connective tissues involved in lifting the eyelid. This muscle weakness or damage is often associated with ageing, and acquired blepharoptosis is more common in older individuals.

How severe the condition is depends on how much the upper eyelid droops. If the eyelid droops over the pupil, it may impact the person's vision and reduce their quality of life. One way to measure the severity of blepharoptosis is using the **margin reflex distance-1** (or **MRD-1**). MRD-1 is the vertical distance from the edge of the upper eyelid to the corneal light reflex, which is the light reflection that appears over or near the center of the pupil when a light is directed straight into the eyes. In a healthy eye, the MRD-1 is about 4 to 5 mm, and a lower value indicates more eyelid droop. It can vary by ethnicity. The lower the value, the more severe the blepharoptosis.

How is acquired blepharoptosis treated?

Until recently, surgery was the main way to treat blepharoptosis. However, there are risks involved with surgery, such as an uneven appearance between the eyes, infections, and

bruising. Because of these risks, surgery might not be the best choice for everyone, especially people with mild blepharoptosis or individuals who do not want to undergo the procedure.

Oxymetazoline 0.1% eye drops are now available in the USA. Studies in the USA have shown that these eye drops help improve blepharoptosis, as measured by MRD-1, and do not cause unwanted side effects when used once a day. These drops provide a less invasive alternative to surgery. However, they are not available in Japan, where surgery is still the main form of treatment.

Who took part in the study?

This study included 336 adults in Japan who had acquired blepharoptosis in one or both eyes.

Individuals who were suitable to take part in the study used oxymetazoline eye drops once or twice a day or a **placebo** (eye drops with no medicine) twice a day for 3 months. Eye drops were used in the morning and evening, and participants who used oxymetazoline once a day used a placebo for the second drop. After 3 months, those already using oxymetazoline continued with the eye drops for a further 3 months. Participants who used placebo eye drops for the first 3 months were switched to using oxymetazoline eye drops either once or twice a day for the next 3 months.

MRD-1 was measured before individuals started using either oxymetazoline or the placebo. After 14 days of treatment, MRD-1 was measured again at various timepoints, including 15 minutes and 2 hour after the morning and 15 minutes after the evening eye drops. The MRD-1 values taken before and after using either oxymetazoline or the placebo were compared to see whether oxymetazoline improved the blepharoptosis. MRD-1 was also measured 43 days, 3 months, and 6 months after treatment began to see how effective oxymetazoline was over time.

Any unwanted side effects that the study investigators thought were directly caused by the treatment, known as **adverse drug reactions**, were recorded throughout the study.

What were the results of the study?

After 14 days of treatment, the investigators found that MRD-1 improved from the start of treatment to 2 hours after the first eye drop, and 15 minutes after the second eye drop with oxymetazoline eye drops more than with the placebo eye drops.

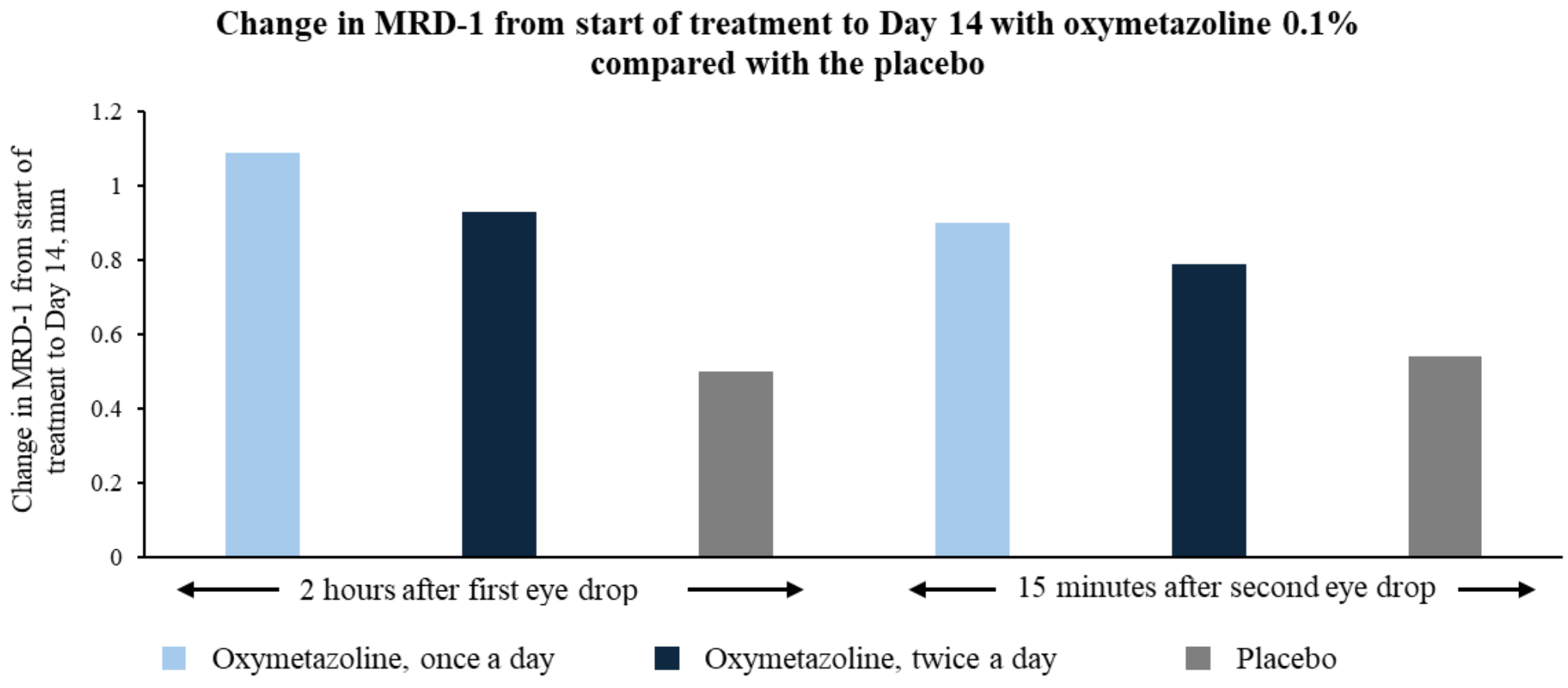

The effects of oxymetazoline on MRD-1 after the first eye drop in the morning lasted for more than 8 hours. In addition, oxymetazoline, used once or twice daily, improved MRD-1 throughout the 6-month study period.

The proportion of individuals experiencing adverse drug reactions was low. In the first 3 months, 0.9% of people using oxymetazoline once daily and 4.5% of people using oxymetazoline twice daily reported adverse reactions compared with 4.5% on placebo. In the next 3 months, no one using oxymetazoline once daily experienced adverse reactions, while 4.6% using it twice daily did. 1.8% of those who switched from placebo to oxymetazoline experienced adverse reactions.

What are the limitations of the evidence?

The evidence is limited because most of the participants (85.7%) were female, which means the effects of oxymetazoline were not tested on many males. Additionally, people with blepharoptosis caused by myasthenia gravis, a common eye condition that leads to acquired blepharoptosis, were not included in the study. By excluding these individuals, it is not possible to say whether oxymetazoline has the same effects on people with acquired blepharoptosis due to myasthenia gravis.

What are the main conclusions of the study?

The results of this study show that in Japanese people with acquired blepharoptosis, oxymetazoline used once or twice a day for 14 days improves eyelid drooping, as measured by MRD-1. The MRD-1 improvements with oxymetazoline lasted for more than 8 hours after the first eye drop was used. The effects of oxymetazoline continued over 6 months of treatment. The proportion of people who experienced adverse drug reactions was low with oxymetazoline.

These results suggest that in Japanese people, oxymetazoline, used once or twice a day, may be a suitable treatment option for acquired blepharoptosis.

Disclosures:

This summary was prepared by Lucy Helas, BSc, of Core (a division of Prime, London, UK). Plain-language services were funded by Santen. The original authors of the full publication were not involved in preparing the summary.
