# Supplementary material for: Six-month efficacy and safety of oxymetazoline hydrochloride 0.1% in Japanese patients with acquired blepharoptosis: a phase 3 study
Source: Jpn J Ophthalmol. 2026 Apr 10;70(4):820–9. doi: 10.1007/s10384-026-01340-5 (PMC13452870; doi:10.1007/s10384-026-01340-5)
Supplement: Supplementary file 4 [file 10384_2026_1340_MOESM4_ESM.pdf]

Table S1 95% CI of the Difference in MRD-1 Change From Baseline Between OMZ 0.1% and Placebo at Each Timepoint During Treatment Period 1

Analysis visit	OMZ 0.1% QD (N=112)	OMZ 0.1% BID (N=112)
**Day 14 (5 minutes after the morning drop)**		
n	110	110
95% CI of difference (OMZ 0.1% vs. placebo)	–0.11304, 0.29659	–0.22469, 0.15751
**Day 14 (15 minutes after the morning drop)**		
n	108	111
95% CI of difference (OMZ 0.1% vs. placebo)	0.25269, 0.66709	0.04279, 0.42730
**Day 14 (2 hours after the morning drop)**		
n	110	110
95% CI of difference (OMZ 0.1% vs. placebo)	0.38922, 0.81275	0.19391, 0.62442
**Day 14 (6 hours after the morning drop)**		
n	109	111
95% CI of difference (OMZ 0.1% vs. placebo)	0.31543, 0.75067	0.02531, 0.46181
**Day 14 (8 hours after the morning drop)**		
n	110	111
95% CI of difference (OMZ 0.1% vs. placebo)	0.33162, 0.74809	0.04895, 0.46114
**Day 14 (5 minutes after the evening drop)**		
n	109	111
95% CI of difference (OMZ 0.1% vs. placebo)	0.20859, 0.64090	0.03557, 0.46344
**Day 14 (15 minutes after the evening drop)**		
n	110	109
95% CI of difference (OMZ 0.1% vs. placebo)	0.15470, 0.61003	0.00481, 0.45400

CI, confidence interval; BID, twice daily; OMZ 0.1%, oxymetazoline hydrochloride 0.1%; QD, once daily
