# Supplementary material for: Six-month efficacy and safety of oxymetazoline hydrochloride 0.1% in Japanese patients with acquired blepharoptosis: a phase 3 study
Source: Jpn J Ophthalmol. 2026 Apr 10;70(4):820–9. doi: 10.1007/s10384-026-01340-5 (PMC13452870; doi:10.1007/s10384-026-01340-5)
Supplement: Supplementary file 5 [file 10384_2026_1340_MOESM5_ESM.pdf]

Table S2 Summary of any-grade AEs by MedDRA preferred term (occurring at a rate of ≥1%): Treatment Period 1

**Patients, n (%)**	**OMZ 0.1% QD (n=112)**	**OMZ 0.1% BID (n=112)**	**Placebo (n=112)**
**Any AE**	28 (25.0)	30 (26.8)	27 (24.1)
**Eye disorders**	5 (4.5)	12 (10.7)	11 (9.8)
Dry eye	2 (1.8)	4 (3.6)	1 (0.9)
Allergic conjunctivitis	2 (1.8)	1 (0.9)	3 (2.7)
Punctate keratitis	1 (0.9)	3 (2.7)	1 (0.9)
Blurred vision	0 (0.0)	1 (0.9)	2 (1.8)
**Infections and infestations**	9 (8.0)	8 (7.1)	5 (4.5)
COVID-19	3 (2.7)	0 (0.0)	2 (1.8)
Nasopharyngitis	2 (1.8)	1 (0.9)	2 (1.8)
**Gastrointestinal disorders**	4 (3.6)	4 (3.6)	3 (2.7)
Abdominal discomfort	0 (0.0)	2 (1.8)	0 (0.0)
Diarrhea	0 (0.0)	0 (0.0)	2 (1.8)
**Injury, poisoning, and procedural complications**	4 (3.6)	3 (2.7)	2 (1.8)
**Nervous system disorders**	2 (1.8)	2 (1.8)	5 (4.5)
Headache	0 (0.0)	1 (0.9)	2 (1.8)
Hypoesthesia	0 (0.0)	0 (0.0)	2 (1.8)
**Immune system disorders**	2 (1.8)	2 (1.8)	1 (0.9)
Seasonal allergy	2 (1.8)	2 (1.8)	1 (0.9)
**Musculoskeletal and connective tissue disorders**	1 (0.9)	1 (0.9)	3 (2.7)

**General disorders and administration site conditions**	1 (0.9)	1 (0.9)	2 (1.8)
**Skin and subcutaneous tissue disorders**	2 (1.8)	0 (0.0)	3 (2.7)
Eczema	0 (0.0)	0 (0.0)	2 (1.8)
**Neoplasms benign, malignant, and unspecified[a]**	1 (0.9)	1 (0.9)	2 (1.8)
**Respiratory, thoracic, and mediastinal disorders**	0 (0.0)	0 (0.0)	3 (2.7)

[a]Including cysts and polyps
AE, adverse event; BID; twice daily; COVID-19, coronavirus disease 2019; MedDRA, Medical Dictionary for Regulatory Activities; OMZ 0.1%, oxymetazoline hydrochloride 0.1%; QD, once daily
