# Supplementary material for: Six-month efficacy and safety of oxymetazoline hydrochloride 0.1% in Japanese patients with acquired blepharoptosis: a phase 3 study
Source: Jpn J Ophthalmol. 2026 Apr 10;70(4):820–9. doi: 10.1007/s10384-026-01340-5 (PMC13452870; doi:10.1007/s10384-026-01340-5)
Supplement: Supplementary file 6 [file 10384_2026_1340_MOESM6_ESM.pdf]

Table S3 Summary of any-grade AEs by MedDRA-preferred term (occurring at a rate of ≥1%): Treatment Period 2

Patients, n (%)	OMZ 0.1% QD (n=110)	OMZ 0.1% BID (n=109)	Placebo/OMZ 0.1% QD (n=55)	Placebo/OMZ 0.1% BID (n=55)
**Any AE**	34 (30.9)	27 (24.8)	14 (25.5)	13 (23.6)
**Eye disorders**	10 (9.1)	11 (10.1)	2 (3.6)	4 (7.3)
Conjunctivitis allergic	2 (1.8)	2 (1.8)	0 (0.0)	2 (3.6)
Conjunctival hemorrhage	3 (2.7)	0 (0.0)	1 (1.8)	1 (1.8)
Dry eye	1 (0.9)	3 (2.8)	0 (0.0)	1 (1.8)
Punctate keratitis	1 (0.9)	1 (0.9)	0 (0.0)	1 (1.8)
Conjunctival hyperemia	0 (0.0)	2 (1.8)	0 (0.0)	0 (0.0)
Vision blurred	0 (0.0)	1 (0.9)	1 (1.8)	0 (0.0)
Vitreous floaters	1 (0.9)	0 (0.0)	0 (0.0)	1 (1.8)
**Infections and infestations**	11 (10.0)	7 (6.4)	4 (7.3)	3 (5.5)
COVID-19	4 (3.6)	2 (1.8)	0 (0.0)	2 (3.6)
Nasopharyngitis	2 (1.8)	0 (0.0)	1 (1.8)	1 (1.8)
Conjunctivitis	2 (1.8)	0 (0.0)	0 (0.0)	0 (0.0)
Dermatophytosis of nail	0 (0.0)	0 (0.0)	1 (1.8)	0 (0.0)

Influenza	0 (0.0)	0 (0.0)	1 (1.8)	0 (0.0)
Tinea pedis	0 (0.0)	0 (0.0)	1 (1.8)	0 (0.0)
**Nervous system**	5 (4.5)	3 (2.8)	0 (0.0)	3 (5.5)
Cervicobrachial syndrome	0 (0.0)	1 (0.9)	0 (0.0)	1 (1.8)
Dizziness	2 (1.8)	0 (0.0)	0 (0.0)	0 (0.0)
Hypoesthesia	0 (0.0)	0 (0.0)	0 (0.0)	2 (3.6)
**Gastrointestinal**	3 (2.7)	4 (3.7)	2 (3.6)	1 (1.8)
Constipation	0 (0.0)	1 (0.9)	1 (1.8)	0 (0.0)
Abdominal pain	0 (0.0)	0 (0.0)	0 (0.0)	1 (1.8)
Gastrointestinal disorder	0 (0.0)	0 (0.0)	1 (1.8)	0 (0.0)
**Injury, poisoning, and procedural**	3 (2.7)	2 (1.8)	2 (3.6)	2 (3.6)
Contusion	2 (1.8)	0 (0.0)	0 (0.0)	1 (1.8)
Injury corneal	0 (0.0)	0 (0.0)	0 (0.0)	1 (1.8)
Ligament injury	0 (0.0)	0 (0.0)	1 (1.8)	0 (0.0)
Thermal burn	0 (0.0)	0 (0.0)	1 (1.8)	0 (0.0)
**Musculoskeletal and connective tissue disorders**	3 (2.7)	2 (1.8)	3 (5.5)	1 (1.8)

Musculoskeletal stiffness	1 (0.9)	0 (0.0)	1 (1.8)	0 (0.0)
Arthralgia	0 (0.0)	0 (0.0)	0 (0.0)	1 (1.8)
Back pain	0 (0.0)	0 (0.0)	0 (0.0)	1 (1.8)
Neck pain	0 (0.0)	0 (0.0)	0 (0.0)	1 (1.8)
Pain in extremity	0 (0.0)	0 (0.0)	1 (1.8)	0 (0.0)
Tenosynovitis	0 (0.0)	0 (0.0)	1 (1.8)	0 (0.0)
**General disorders and administration site conditions**	2 (1.8)	1 (0.9)	0 (0.0)	1 (1.8)
Malaise	0 (0.0)	0 (0.0)	0 (0.0)	1 (1.8)
**Skin and subcutaneous tissue disorders**	2 (1.8)	0 (0.0)	2 (3.6)	0 (0.0)
Dermatitis	0 (0.0)	0 (0.0)	1 (1.8)	0 (0.0)
Eczema	0 (0.0)	0 (0.0)	1 (1.8)	0 (0.0)
**Ear and labyrinth disorders**	1 (0.9)	0 (0.0)	1 (1.8)	0 (0.0)
Sudden hearing loss	0 (0.0)	0 (0.0)	1 (1.8)	0 (0.0)
**Immune system disorders**	0 (0.0)	1 (0.9)	1 (1.8)	0 (0.0)
Seasonal allergy	0 (0.0)	1 (0.9)	1 (1.8)	0 (0.0)

**Neoplasms benign, malignant, and unspecified (including cysts and polyps)**	1 (0.9)	0 (0.0)	1 (1.8)	0 (0.0)
Skin papilloma	0 (0.0)	0 (0.0)	1 (1.8)	0 (0.0)
**Respiratory, thoracic, and mediastinal**	0 (0.0)	0 (0.0)	1 (1.8)	1 (1.8)
Allergic pharyngitis	0 (0.0)	0 (0.0)	0 (0.0)	1 (1.8)
Rhinitis allergic	0 (0.0)	0 (0.0)	1 (1.8)	0 (0.0)

AE, adverse event; BID; twice daily; COVID-19, coronavirus disease 2019; MedDRA, Medical Dictionary for Regulatory Activities; OMZ 0.1%, oxymetazoline hydrochloride 0.1%; QD, once daily
