# Supplementary material for: Six-month efficacy and safety of oxymetazoline hydrochloride 0.1% in Japanese patients with acquired blepharoptosis: a phase 3 study
Source: Jpn J Ophthalmol. 2026 Apr 10;70(4):820–9. doi: 10.1007/s10384-026-01340-5 (PMC13452870; doi:10.1007/s10384-026-01340-5)
Supplement: Supplementary file 7 [file 10384_2026_1340_MOESM7_ESM.pdf]

Fig. S1. Subgroup analysis by (a) sex, (b) age, and (c) baseline MRD-1 value of the MRD-1 change from baseline to Day 14 (2 hours after the morning drop)

a

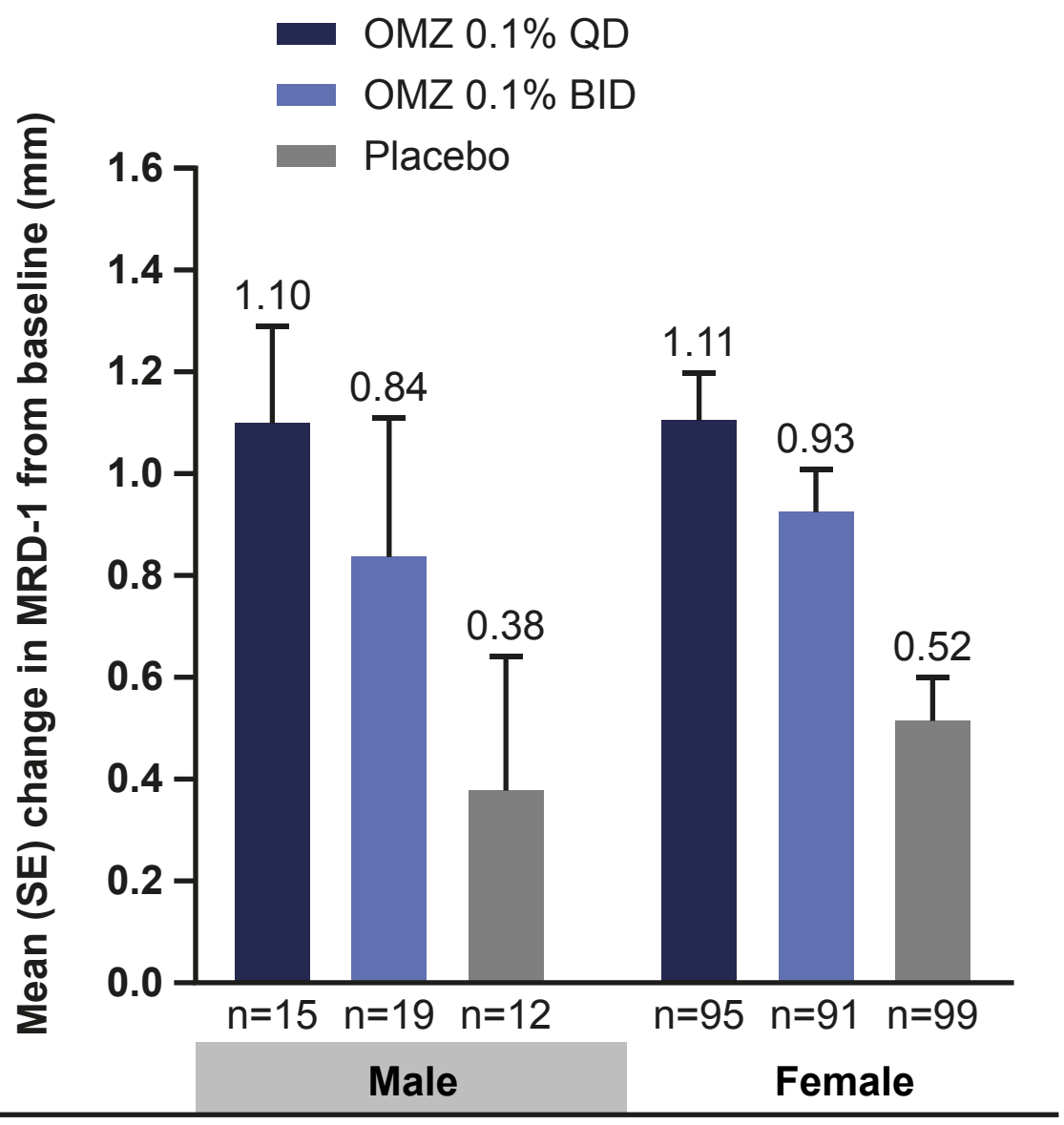

	Male OMZ 0.1% QD	Male OMZ 0.1% BID	Female OMZ 0.1% QD	Female OMZ 0.1% BID
Mean (SD) difference from placebo	0.71 (0.81)	0.46 (1.09)	0.59 (0.80)	0.41 (0.76)
95% CI of difference	0.07, 1.36	–0.36, 1.28	0.36, 0.81	0.19, 0.63

b

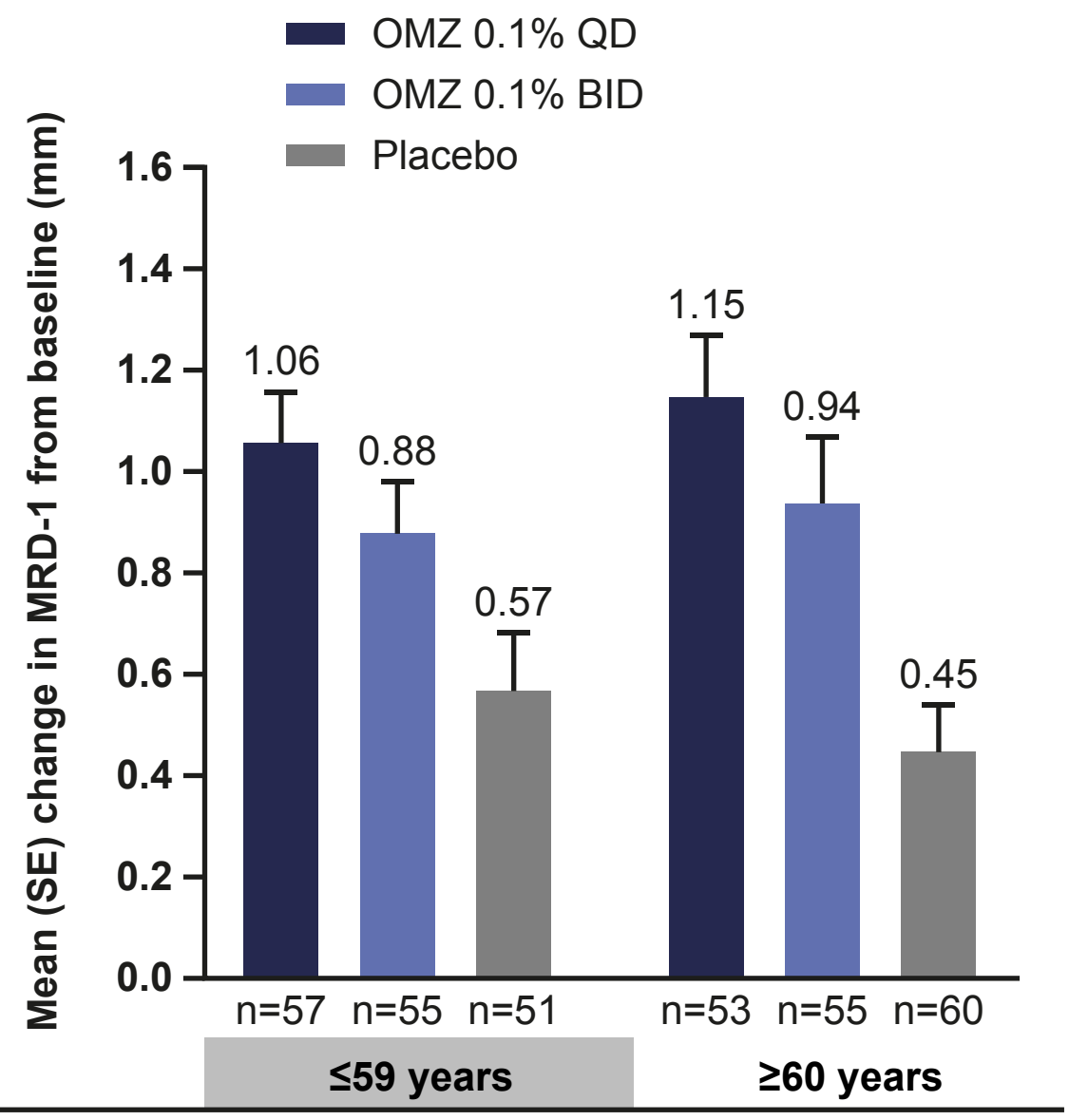

	≤59 years OMZ 0.1% QD	≤59 years OMZ 0.1% BID	≥60 years OMZ 0.1% QD	≥60 years OMZ 0.1% BID
Mean (SD) difference from placebo	0.49 (0.80)	0.31 (0.76)	0.71 (0.81)	0.49 (0.86)
95% CI of difference	0.18, 0.79	0.02, 0.61	0.41, 1.01	0.18, 0.81

c

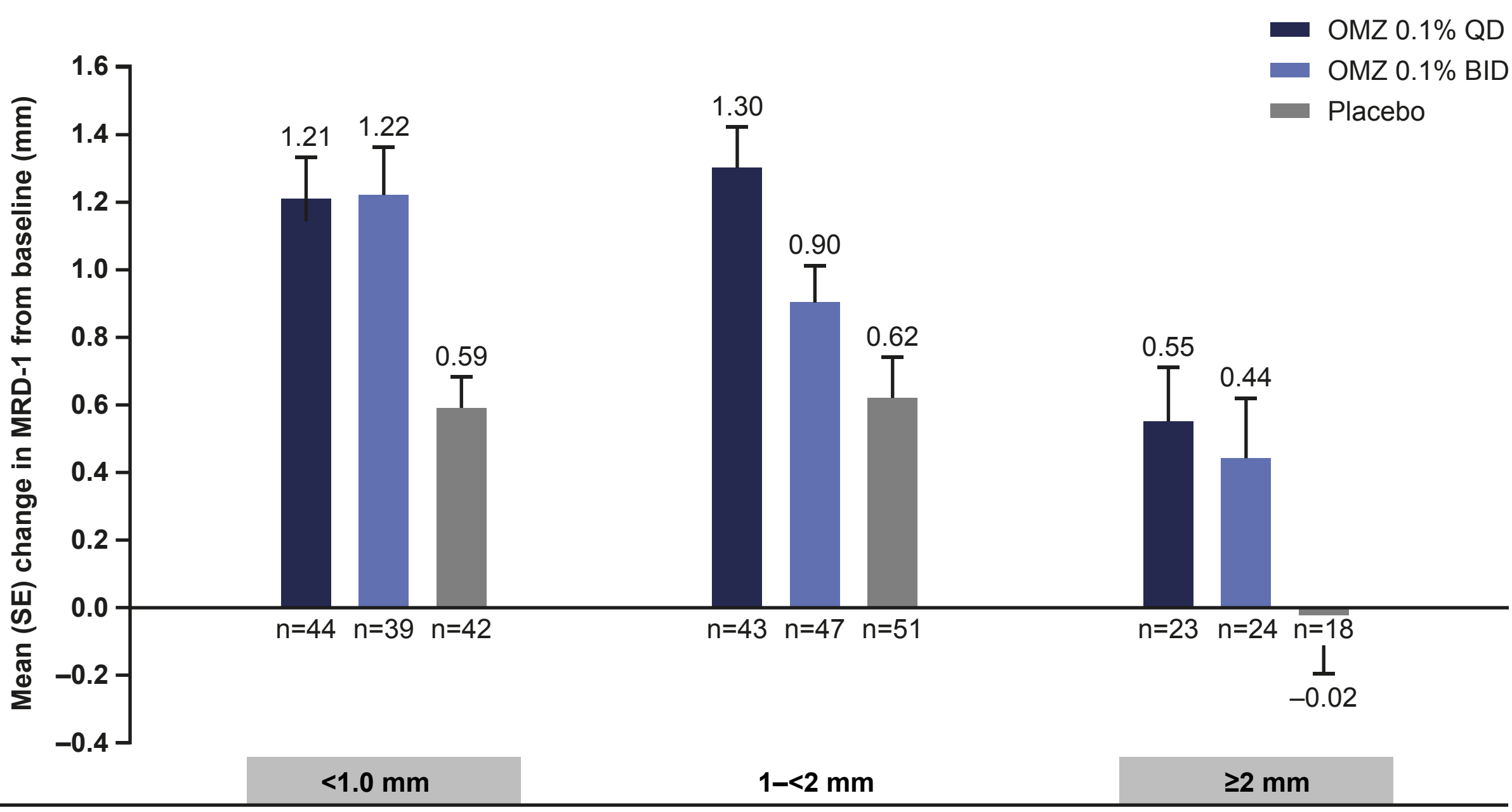

	<1.0 mm OMZ 0.1% QD	<1.0 mm OMZ 0.1% BID	1–<2 mm OMZ 0.1% QD	1–<2 mm OMZ 0.1% BID	≥2 mm OMZ 0.1% QD	≥2 mm OMZ 0.1% BID
Mean (SD) difference from placebo	0.62 (0.69)	0.63 (0.73)	0.68 (0.82)	0.28 (0.79)	0.57 (0.77)	0.46 (0.83)
95% CI of difference	0.32, 0.91	0.31, 0.95	0.34, 1.01	–0.04, 0.60	0.08, 1.06	–0.06, 0.99

Subgroup analysis findings should be interpreted with caution due to possible regression or selection effects from screening to baseline

BID, twice daily; CI, confidence interval; MRD-1, marginal reflex distance-1; OMZ, oxymetazoline hydrochloride 0.1%; QD, once daily; SE, standard error
